# Fever Physiologically Considered; Considerations on Yellow Fever, Typhus Fever, Plague, Cholera, and Sea-Scurvy; Also the Questions of Contagion and the Quarantine Laws; with an Address to the Public, &c., on the Popular Treatment of Cholera

**Published:** 1847-01

**Authors:** 


ART. V.—*Fever physiologically considered; considerations on Yellow Fever, Typhus Fever, Plague, Cholera, and Sea-Scurvy; also the Questions of Contagion and the Quarantine Laws; with an Address to the Public, &c. on the popular Treatment of Cholera.* By DAVID M'CONNELL REED, Esq. Licentiate of Medicine &c.—*London,* 1846. Small 8vo, pp. 262.

DR. M'CONNELL REED has taken great pains with his book, or at least with that part of it which refers to fever. The history and whole pathology of the different forms of fever are considered at length and in minute detail; but, we are sorry to say, most unsatisfactorily.

With regard to the second part of the work we cannot express any favorable opinion. It is devoted to a consideration of the nature, cause, and treatment of epidemic yellow fever, typhus fever, plague, cholera, and sea-scurvy, the latter disease being most erroneously considered identical with the others mentioned.

The appendix is an address "to the humane public, and to the honorable members of the medical profession" on the near advent of Asiatic cholera, and on the method of treating it. The author appears to be possessed of some talent, but it is sadly obscured by singular religious expressions, and by curiously new terms. For example, in his physiological estimate of the symptoms which depend on disorder of the brain and nervous system, Dr. Reed observes,—

"Fainting or syncope depends on suspended or probably retrograde action of the capillary vessels of the fibro-serous membrane of the brain; whence result, loss of the mento-organic faculty of sensibility, and of the animo-organic function of sensation; the mento-muscular faculty of mobility-at-will, and of the animo-organic function of voluntary motion; the total suspension of the animo- or mento-organic faculties, &c." (p. 57.)

Doubtless Dr. Reed uses these terms with a definite meaning, but we think he ought to have added a dictionary or glossary to his book.

With regard to the prophetic and religious expressions of the author, we shall content ourselves with quoting the following piece of presumption:

"The author confidently predicts a dreadful visitation of the cholera at the close of summer, or at the beginning of autumn, most likely in the month of August, to be succeeded by a desolating outbreak of typhus fever. He moreover predicts that those who will fall victims to these diseases are the *very highest* and the *very lowest* classes of society—those who, in consequence of their imprudence and profligacy, have induced want and starvation, with its accompanying miseries; and those who, in consequence of their avarice, gluttony, intemperance, and inhumanity, have induced an unhealthy, plethoric state of the system, coupled with an *uneasy conscience.* The author thinks he could go farther, and point out some of the individuals on whom this judgment will fall; but he forbears, leaving *secret things to the Allwise Judge, and Disposer* of all *events* both in heaven and earth." (pp. 225-6.)

Dr. Reed further trusts much, as we learn, to "the efficacy of faithful prayer, vested in the church," relief in the coming troublous times.

Our author seems to be a man mainly of *three* books; Thomas's 'Practice of Physic;' Hooper's 'Medical Dictionary;' and the 'Bible.' The latter affords mottoes; the first two numerous extracts. We cannot approve of the mixture of sacred and profane learning; the assumption of the power of prophecy, the judging of others; and the praying in public (on the sheets of his book) displayed by Dr. Reed. He should

"heartily thank God" in his closet and not on the wings of the press; and he should remember that divine law, "judge not, that ye be not judged," and not hint dark suspicions respecting his neighbours. There are evidently some elements of power in Dr. Reed's mind, but to use them aright he must undergo a course of mental discipline, or at least should learn to restrain his philological vagaries and check the exuberance of his religious imaginings.

